# Applying Behavioural Incentives to Increase Adherence to Maintenance Treatment

**DOI:** 10.21315/mjms2018.25.6.14

**Published:** 2018-12-28

**Authors:** Saeid Komasi, Mozhgan Saeidi, Payam Sariaslani, Ali Soroush

**Affiliations:** 1 Clinical Research Development Centre, Imam Reza Hospital, Kermanshah University of Medical Sciences, Kermanshah, Iran; 2 Cardiac Rehabilitation Centre, Imam Ali Hospital, Kermanshah University of Medical Sciences, Kermanshah, Iran; 3 Neurology Department, Imam Reza Hospital, Kermanshah University of Medical Sciences, Kermanshah, Iran; 4 Lifestyle Modification Research Centre, Imam Reza Hospital, Kermanshah University of Medical Sciences, Kermanshah, Iran

**Keywords:** behaviour modifications, token reinforcement, drug abuse treatment centres, medication compliance

## Abstract

A significant portion of the various communities, especially developing countries, is involved in drug abuse and receive formal drug treatments. Although the benefits of available therapeutics such as methadone maintenance treatment (MMT) for controlling infectious diseases have been confirmed, treatment failure has been seen in a large range of the patients. This review addresses the importance of a less attentive behavioural approach in reducing treatment withdrawal. The executive protocol, the outcomes and challenges, and the benefits of this approach are debatable.

## Introduction

Currently, methadone maintenance treatment (MMT) delivery by formal centres is one of the most commonly used therapeutics for controlling and reducing the morbidity and mortality related to drug abuse (1). Although the benefits of this therapeutic approach to the control of infectious diseases such as AIDS and hepatitis have been confirmed (2), current reports indicate that the treatment failure and abuse resumption in the significant range of patients (3). The treatment failure and frustration and inability to regain health, especially if the drug slip occurs frequently, the patient encountered a worse condition. At this stage, the patient may commit a high-risk behaviour without any prior history (4). Even, the person may use more harmful substances. Naturally, these risky behaviours lead to a set of adverse personal, familial, and social consequences and inflict much damage to the health system of the country (4).

Based on these considerations, it seems necessary to adopt strategies to increase adherence to the MMT (3). The significance of the issue is more highlighted if we know that many portions of the various communities, especially developing countries, are addicted and currently receive drug treatments. Drug abuse, specifically dependence on opiates, is prevalent 6.4%–12% in Asian countries (5). For example, about 4 million Iranians are addicted and 83% of them are under conventional treatments MMT (6).

## Behavioural Incentives in Drug Addiction Treatment

Among all the addicted patients, many have experience at least one unsuccessful drug therapy (7). After three years, the rate of treatment retention is reduced by 20% (8). In order to control this dilemma and to increase adherence to conventional treatment, the use of behavioural incentives approach is a recommended method (9). In behavioural psychology, incentive/reinforcement is a consequence that will strengthen an organism’s future behaviour whenever that behaviour is preceded by a specific antecedent stimulus (10). A behavioural incentive programme can be implemented by the staff of each MMT centres as follows: Firstly, the health teams at each MMT centres receive training on the designing and implementation of behavioural incentives. These training can be provided by experienced instructors and specialists from the Ministry of Health. Then, the staff of each health centre under the supervision of a doctor can take action to design the chart of incentives. These experts can be modeled from similar groups programme such as narcotics anonymous (NA) to design of the behavioural incentive programme.

## Modeling Based on Successful Parallel Programmes

In the NA avoidance-based programme, each patient as soon as he or she leaves the drug is directly supported by a person who has already entered the discontinuation phase. Meanwhile, the patient participates in daily group meetings (at least three times a week) and is encouraged by other audiences verbally and mentally (11–12). In addition, for each year of drug discontinuation, a birthday party is held and a donation is given to the patient. This could reminds the patient the number of years of drug discontinuation. This behavioural incentives process in NA groups has been able to play an effective role in controlling substance abuse. The more important note is that the pattern of abandoning drug in these people is persisting and they try to go through the health twelve-steps defined in the NA protocol (12–13). Clearly, in this system, the patient is not abandoned by other patients and is constantly receiving a variety of behavioural incentives. This routine can be a good model for the treatment team of the MMT centres.

## How to Implement a Behavioural Incentive Programme

At the first step, a protocol can be prepared for patients and provided it to the patient as soon as the treatment starts. Our proposed projects are as follows: (i) provide a behavioural token to stabilise dosage (TSD) for the patients. The TSD is provided to the patient due to dose stabilisation during one month (i.e., not increasing the dose), (ii) a behavioural token to reduce dosage (TRD) can be provided to the patient due to each mL decreases the dose of the drug, (iii) if receive six TSD or three TRD by the patient, he/she can benefit from a 5% reduction in total treatment costs. This approach can greatly enhance the patient’s efforts to stop drug abuse and create a stable healthy behaviour. In this regard, previous studies have pointed to the role of financial incentives in adopting healthy behaviours (14, 15). Of course, in order to prevent the financial loss of the health system teams and prevents their opposition to the implementation of the plan, (iv) it is advisable to pay the discount in the form of a subsidy to the MMT centres. This subsidy can be provided directly by the Ministry of Health or the Welfare Organisation and the medical sciences universities (16). Thus, both patients and healthcare personnel have enough motivation to implement this programme.

## Outcomes and Challenges

Ultimately, the treatment team needs to repeatedly provide verbal incentives to the patients and the social worker of the centre should help to continue the patients’ health behaviour through telephone or face-to-face follow-ups. However, there are probably some limitations in spite of the above benefits. For example, the patients with fatal comorbidities and end-stage people may not pay much attention to the behavioural incentives due to lack of sufficient motivation to survive. This method is not effective for those who have entered treatment due to family and social pressures. Based on the theory of the stages of change, these patients are at the pre-contemplation stage, and even direct methods do not help them a lot (17). In addition, antisocial and paranoid characters are less likely to use this approach because of pessimism and communication problems. In general, the pathology of behavioural tokens and economics needs to be considered exactly (18).

## Advantages and Recommendations

Although the behavioural incentives approach may not be useful to a number of addicted patients, it seems that the implementation of this targeted system or similar patterns can be effective in increased adherence to MMT and control the harmful effects of re-abuses (9). On the other hand, drug addicts have a specific deficit in negative reinforcement learning (19). Therefore, it is better to use positive reinforcement methods in order to change their behaviour. Taking into account the sociocultural context of the communities and the case formulation of each patient, this approach can be well applied by drug abuse treatment professionals.

## Conclusions

In order to increase adherence to MMT and drug addiction treatments, the use of the behavioural incentives approach is a recommended method. The behavioural incentive programme can be implemented by an MMT centre staff in the proposed format of this review. The behavioural token to stabilise dosage (TSD) and the behavioural token to reduce dosage (TRD) can be provided effectively to the patients. Despite the potential challenges and limitations, this approach can be well applied by drug abuse treatment professionals.
